# Study of Utility of Basic Arterial Blood Gas Parameters and Lactate as Prognostic Markers in Patients With Severe Dengue

**DOI:** 10.7759/cureus.24682

**Published:** 2022-05-03

**Authors:** Manoj Gupta, Nipun Agrawal, Sanjeev K Sharma, Azmat Kamal Ansari, Tariq Mahmood, Lalit Singh

**Affiliations:** 1 Biochemistry, Shri Ram Murti Smarak Institute of Medical Sciences, Bareilly, IND; 2 Community Medicine, Shri Ram Murti Smarak Institute of Medical Sciences, Bareilly, IND; 3 Community Medicine, Shri Ram Murti Smarak institute of Medical Sciences, Bareilly, IND; 4 Pulmonary Medicine, Shri Ram Murti Smarak Institute of Medical Sciences, Bareilly, IND

**Keywords:** prognostic markers in severe dengue, severe dengue, prognostic marker, lactate, basic arterial blood gas parameters

## Abstract

Background

The importance of prognostication in critical care cannot be over-emphasized, especially in the context of diseases like dengue, as their presentation may vary from mild fever to critical life-threatening illness. With the help of prognostic markers, it is possible to identify patients at higher risk and thus improve their outcome with timely intervention. Basic arterial blood gas (ABG) parameters, i.e., potential of hydrogen (pH), partial pressure of oxygen (PO2), partial pressure of carbon dioxide (PCO2) and bicarbonate are useful parameters, especially in critical care medicine as they are known to vary with the severity of illness. Hyperlactatemia is often referred to as a “powerful predictor of mortality”. Basic ABG parameters and lactate have been used as an essential prognostic modality in critically ill patients for decades; however, the evidence remains limited for their role as prognostic markers in patients with severe dengue.

Method

We carried out an observational retrospective cohort study comprising 163 patients with severe dengue, admitted between July 2021 and November 2021 at Medical Intensive Care Unit (MICU) of Shri Ram Murti Smarak Institute of Medical Sciences (SRMS IMS), Bareilly, Uttar Pradesh, India. Basic ABG parameters and lactate levels at the time of admission to MICU were compared between survivor and non-survivor groups of patients with severe dengue in order to evaluate their prognostic utility as predictors of mortality.

Results

pH (p<0.0001), PO2 (p=0.01) and bicarbonate (<0.0001) levels were significantly lower, while PCO2 (p=0.002) and lactate (p<0.0001) levels were significantly higher in non-survivor group as compared to survivor group. Lactate was found to be the best prognostic marker with Area Under the Curve (AUC) of 88.7% on Receiver Operating Characteristics (ROC) analysis.

Conclusion

Basic arterial blood gas parameters and lactate can be used as feasible prognostic markers in patients with severe dengue.

## Introduction

Prognostication plays a vital role in patient care, especially in critical care medicine. It prevents discordant expectations about patient outcome between patients’ attendants and health care professionals [1]. It not only allows physicians to identify patients at higher risk of adverse outcomes and plan timely interventions, but also helps in the judicious management of resources [2]. A number of different models for the prognostication of critically ill patients have been proposed but a simpler and more accurate prognostic marker is still awaited especially for patients with dengue.

Dengue fever, though categorized as “neglected tropical disease”, has been a major public health concern, especially in the Indian subcontinent. In the last two decades, the incidence of dengue in India has increased to about 15 per million people annually [3]. Dengue virus, a positive-stranded RNA-containing virus, belongs to Flavivirus species, genus Flavivirus, family Flaviviridae. Clinical presentation of dengue fever may range from mild fever (classical dengue fever) to complicated syndromes (like dengue hemorrhagic fever, and dengue shock syndrome) [4]. Thus, prognostication of the severity of illness is of paramount importance in the management of these patients.

Basic arterial blood gas (ABG) parameters, i.e., potential of hydrogen (pH), partial pressure of oxygen (PO2), partial pressure of carbon dioxide (PCO2) and serum bicarbonate are useful tools in prognostication. They are known to fluctuate with of severity of illness and thus are used in the prediction of mortality in critical care units [5-7]. Lactate, a by‑product of anaerobic metabolism, is considered a marker of tissue hypoxia. Hyperlactatemia is often referred to as “powerful predictor of mortality” as it is an indicator of anaerobic stress [8]. In critically ill patients, it may serve as a biochemical marker of poor prognosis [8-11]. ABG parameters and lactate have been used as essential diagnostic criteria in critical care medicine. These have also been used to assess the effectiveness of treatment modality in critically ill patients for decades [5-11]. However, evidence remains scarce on the role of these parameters as readily available, cost-effective prognostic markers in patients with severe dengue. This study was, therefore, designed to evaluate the utility of basic ABG parameters and lactate as prognostic markers in patients with severe dengue.

## Materials and methods

An observational retrospective cohort study, utilizing data from patients admitted in our hospital from 1st of July 2021 to 30th of November 2021 was conducted after obtaining approval from our Institutional Ethics Committee (IEC), Shri Ram Murti Smarak (SRMS) Institute of Medical Sciences (IMS), Bareilly. Dengue patients admitted (either directly from the emergency department or transferred from other wards) to our medical intensive care unit (MICU) for various critical illnesses (presence of warning signs according to WHO criteria for dengue) were screened for their eligibility as study participants [12]. The patients with severe dengue admitted in the MICU of our tertiary care super specialty hospital (SRMS IMS, Bareilly in Rohilkhand region of Uttar Pradesh, India) during this duration were included in the study. Data were retrieved from patients’ case files as well as our hospital and laboratory information systems (HIS and LIS), version 3. The autogeneous HIS and LIS were developed by SRMS College of Engineering, Technology & Research, Bareilly in 2017. Patients’ clinical profile and laboratory results of the first arterial blood sample collected after admission at MICU were recorded in specially designed pro forma. The analysis of basic ABG parameters (pH, PO2, PCO2 and bicarbonate) and arterial blood lactate of all patients included in the study was done following the standard operating procedure on Radiometer ABL800 Flex (Radiometer, Copenhagen, Denmark) analyzer installed in our MICU.

The following criteria (based on World Health Organization (WHO) criteria for diagnosis of probable, confirmed and severe cases of dengue) were used to define the study participants as patients with severe dengue [12].

Criteria for diagnosis of dengue: Patients with probable dengue (fever plus any two of the following: aches and pain, nausea and/or vomiting, rash, leucopenia, or presence of any warning signs), confirmed with laboratory tests (NS1 antigen, high-titer levels of IgG or positive IgM).

Criteria for diagnosis of severe dengue: Dengue patients with at least one of the following: 1) Decompensated or compensated shock or respiratory compromise due to severe plasma leakage, 2) Severe bleeding that required intervention, 3) Severe organ involvement such as severe hepatitis, acute kidney injury, myocarditis or encephalopathy.

The exclusion criteria were as follows: patients with positive real-time reverse transcription-polymerase chain reaction (RT-PCR) for Coronavirus disease (COVID-19), those with incomplete data (clinical profile or laboratory test results) for further analysis, who were not willing to comply with standard treatment protocol of our hospital or took discharge against medical advice.

Study participants were classified according to age, gender, and acid-base disorders. The following reference ranges were used for the diagnosis of acid-base disorders: pH: 7.35-7.45, PCO2: 35-45 mmHg and bicarbonate 22-26 mEq/L [13]. The study population was also divided into survivors and non-survivor groups, on the basis of their outcome (survival or death) during their stay at MICU in order to evaluate the utility of basic ABG parameters and lactate in predicting mortality of patients with severe dengue.

Data were represented as frequency (percentage), mean ± standard deviation, and median (interquartile range). Linearity of all quantitative data was assessed using Kolmogorov-Smirnov analysis and tests of statistical significance (Student’s unpaired t-test or Mann-Whitney-U test) were used depending upon the data type. Chi-square test/Fischer exact test was used to assess the significance of difference in frequency distribution. Receiver operating characteristics (ROC) curve analysis was used to assess the ability of basic ABG parameters and lactate to predict mortality. Odd’s ratio was calculated considering the 95% confidence interval. SPSS software, version 19.0 (Statistical Package for the Social Sciences Inc, Chicago, IL, USA) was used to carry out statistical analysis. The significance level of <0.05 was considered as statistically significant.

## Results

Out of 209 critically ill patients with dengue admitted from 1st of July 2021 to 30th of November 2021, 163 patients with severe dengue were chosen for the cohort on the basis of our inclusion (based on WHO criteria for diagnosis of severe dengue) and exclusion criteria. Out of these 163 patients, 114 (69.94%) were shifted out of the MICU for further recovery (survivors) while 49 (30.06%) expired despite the same treatment as well as all resuscitative measures (non-survivors). The demographic profiles of enrolled candidates are shown in Table 1 and Table 2. Sixty (36.81%) participants were female, while the rest 103 (63.19%) were male. The youngest patient was 17 years old while the oldest patient was 84 years old. The median age of patients was 43.7 years. On comparing the demographic profiles of study groups, participants were found to be matched for age and gender.

**Table 1:** Gender distribution of study subjects

Characteristics		Total	Survivors	Non-survivors	X^2^	Odd’s ratio	95% C.I.		p-value
							Low	High	
Gender	Female	60	42	18	0.001	1.005	0.502	2.012	0.567
	Male	103	72	31					

**Table 2:** Age distribution of study subjects

Characteristics		Outcome	N	Mean	Std. Deviation	Std. Error Mean	t	p-value
Age (in Years)		Survivor	114	42.74	16.23	1.52	-1.205	0.23
		Non-survivor	49	46.08	16.40	2.34		

We observed that 40 (24.54%) patients (37 in survivors and three in non-survivors) had no acid-base disturbances, while 123 patients had either acidosis [n=111 (68.10%)] or alkalosis [n=12 (7.36%)]. There were nine cases of alkalosis while 68 cases of acidosis in the survivor group while three cases of alkalosis while 43 cases of acidosis in the non-survivor group. Further details of the frequency of acid-base disorders in study subjects are summarized in Table 3. A significant difference was noted in the frequency of various acid-base disorders between the study groups (p<0.0001).

**Table 3:** Frequency of acid-base disorders in study subjects

Acid-Base Disorder	Total	Survivors	Non-survivors	X^2^	p-value
Compensated respiratory acidosis and metabolic alkalosis	03	01	02	52.785	<0.0001
Compensated respiratory alkalosis Metabolic acidosis	09	04	05		
Compensated respiratory acidosis	02	01	01		
Mixed Acidosis	23	06	17		
Mixed alkalosis	01	01	0		
Normal	40	37	03		
Partially compensated metabolic alkalosis	01	0	01		
Partially compensated respiratory alkalosis	01	01	0		
Partially compensated Metabolic acidosis	33	19	14		
Uncompensated metabolic acidosis	09	06	03		
Uncompensated Metabolic alkalosis	04	04	0		
Uncompensated respiratory acidosis	04	03	01		
Uncompensated respiratory alkalosis	01	01	0		
Indeterminate	32	30	02		

**Figure 1:**
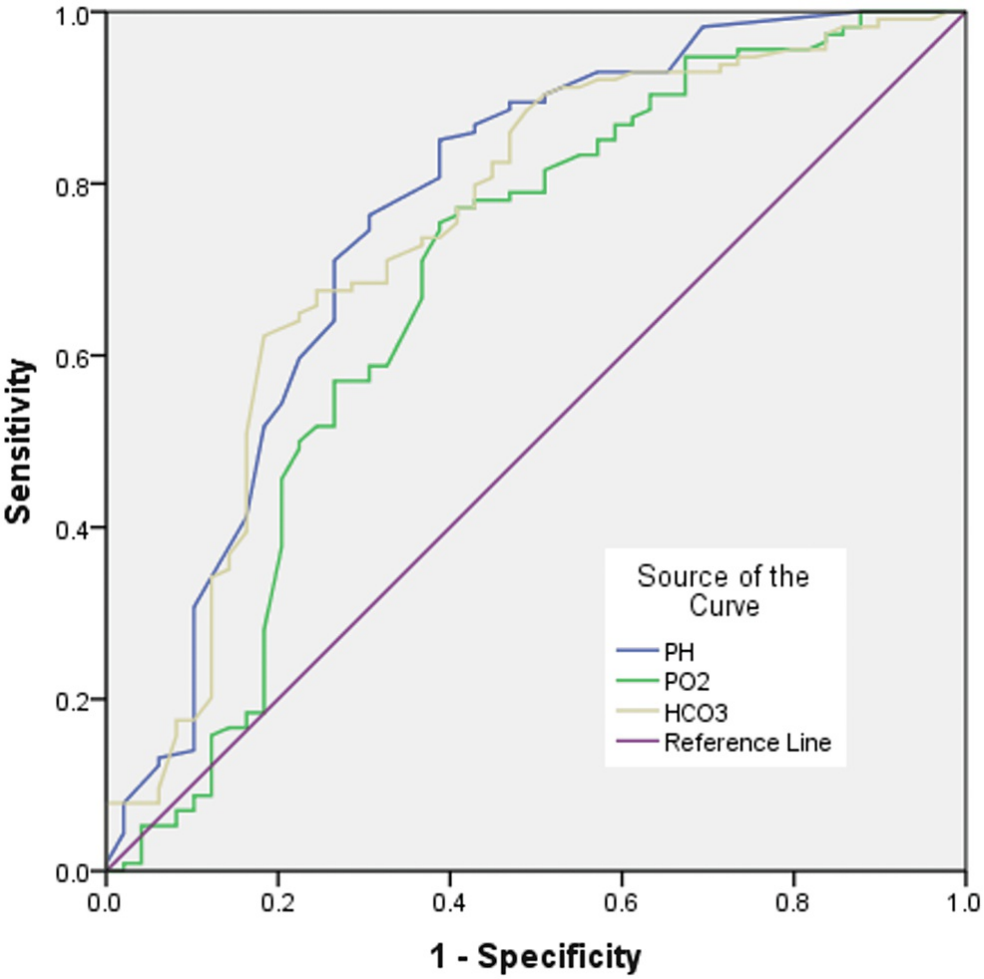
ROC curve of pH, PO2 and Bicarbonate Diagonal segment in the image is produced by ties. HCO3=Bicarbonate PO2=PO_2_

**Figure 2:**
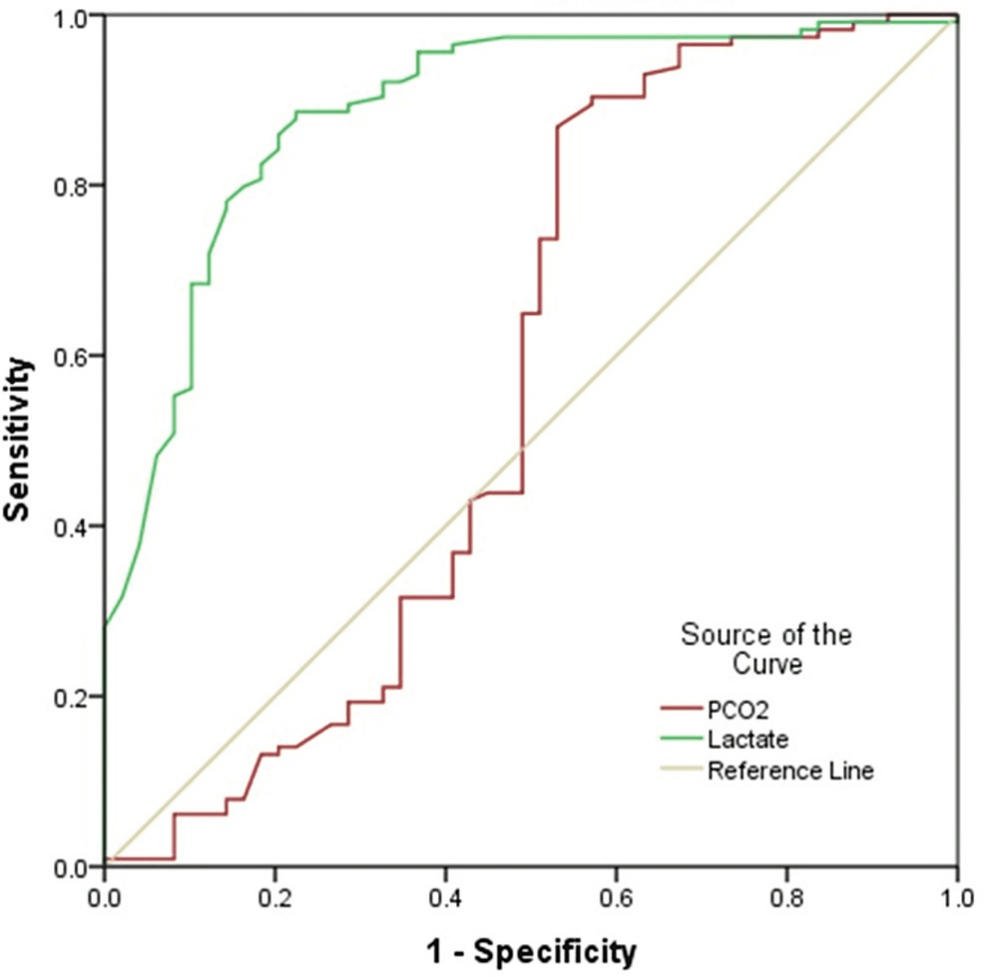
ROC curve of PCO2 and Lactate Diagonal segment in the image is produced by ties. PCO2=PCO_2_

Results of the comparison of basic ABG parameters and lactate are shown in Table 4. In comparison between survivor and non-survivor groups, pH (p<0.0001), PO_2_ (p=0.01) and bicarbonate (<0.0001) were significantly higher in survivors, while PCO_2_ (p=0.002) and lactate (p<0.0001) were significantly higher in the non-survivor group. Table 5 along with Figure 1 and Figure 2 demonstrates the results of ROC curve analysis of various components to assess predictors of mortality.

**Table 4:** Comparison of basic ABG parameters and lactate in study subjects #data expressed in median (interquartile range) to compare using the Mann-Whitney U test ABG: Arterial Blood Gas

Characteristics	Outcome	N	Mean	Std. Deviation	Std. Error Mean	T	p-value
PH	Survivor	114	7.35	0.10	0.01	6.977	<0.0001
	Non-survivor	49	7.18	0.22	0.03		
PCO_2_	Survivor	114	37.13	9.93	0.93	-3.209	0.002
	Non-survivor	49	45.86	24.90	3.56		
PO_2_	Survivor	114	98.30	37.58	3.52	2.599	0.01
	Non-survivor	49	82.41	54.46	7.78		
Bicarbonate	Survivor	114	21.45	5.25	0.49	5.639	<0.0001
	Non-survivor	49	16.07	6.30	0.90		
Lactate^#^	Survivor	114	1.3 (0.9-2.2)	12.50	1.17	-2.481	<0.0001
	Non-survivor	49	07 (3.5-9.6)	5.79	0.83		

**Table 5:** ROC curve analysis of basic ABG parameters and lactate as prognostic markers #AUC for predicting mortality below cut off value $AUC for predicting mortality above cut off value ^a^Under the nonparametric assumption ^b^Null hypothesis: true area = 0.5

Test Result Variable(s)	Area	Std. Error ^a^	Asymptotic Sig. ^b^	Asymptotic 95% Confidence Interval		Cut off	Sensitivity (%)	Specificity (%)
				Lower Bound	Upper Bound			
PH	0.766#	0.045	<0.0001	0.679	0.854	7.455	7.9	98
PO_2_	0.683#	0.050	<0.0001	0.584	0.782	80.1	75.4	61.2
Bicarbonate	0.747#	0.045	<0.0001	0.659	0.835	21.9	62.3	81.6
PCO_2_	0.568$	0.059	0.172	0.453	0.682	44.1	86.6	46.9
Lactate	0.887$	0.029	<0.0001	0.829	0.944	2.65	82.5	81.6

ROC curve analysis was performed to assess the prognostic capacity of basic ABG parameters and lactate in predicting the mortality of the patients with severe dengue. Lactate was found to be the best prognostic parameter with area under the curve (AUC) of 88.7%. The sensitivity and specificity of lactate was found to be 82.5% and 81.6% respectively at the cut-off value of 2.65 units.

## Discussion

The quest for accurate identification of patients with severe dengue who have the potential to further deteriorate after admission is still inadequate [14]. Platelets count has been used for long as a parameter to keep track of the progression of dengue severity, but it has certain limitations [15]. Traditional prognostic markers such as symptoms (pattern of fever, nausea, vomiting, aches and pain, etc.), signs (positive tourniquet test, respiratory distress, features suggestive of plasma leakage, bleeding, fluid accumulation, etc.) and laboratory tests (leukopenia, organ function tests suggestive of severe organ involvement, etc.) remained sensitive indicators of underlying physiological disturbance but these are complex and thus subjected to errors in interpretation [12,14-21]. The World Health Organization (WHO) revised dengue case classification criteria for differentiation between classic dengue fever and severe dengue (that includes severe bleeding, shock, respiratory compromise as well as severe organ involvement such as hepatitis, kidney injury, myocarditis or encephalopathy) [12]. Although the revised criteria are more sensitive to the diagnosis of severe dengue, there remain issues with its applicability [12,14,16]. Thus, simpler, quick, easily available, and cost-effective prognostic parameters are still being sought [22].

Acid-base disorders and serum bicarbonate are well-validated markers of pathophysiological disturbances caused by critical illnesses and thus are correlated with mortality [5-11]. Elevated blood lactate levels associated with metabolic acidosis are common among critically ill patients with systemic hypoperfusion and tissue hypoxia [8]. Several studies have suggested that blood lactate concentration has prognostic value in patients requiring critical care [8-11]. Few studies have confirmed the role of peripheral venous lactate levels in predicting the severity of dengue [10-11]. Despite easy availability of rapid blood gas and lactate measurements in critical care setup, the data evaluating these parameters in the prognosis of critically ill dengue patients are limited. Based on our cohort, predictors of death in patients with severe dengue were: basic ABG parameters, acid-base disorders (especially acidosis), and lactate levels. We observed statistically significant differences in pH, pO2, pCO2 and bicarbonate levels between the survivor and non-survivals groups. Lower pH, PO2 and bicarbonate, while higher PCO2 and lactate levels were found to be associated with mortality in patients with severe dengue. Other studies focused on studying the factors associated with death among severe dengue patients have shown similar results but only few have studied these basic ABG parameters and fewer have used WHO classification in demarcating cases as patients with severe dengue [10-12,16-21,23-24]. Md-Sani et al. concluded that serum bicarbonate and serum lactate are independent predictors of death among severe dengue patients [23]. However, on multivariate regression analysis, we found that none of the individual basic ABG parameters were independent predictors of mortality in our study population. We propose that the use of multivariate regression analysis in order to evaluate the utility of individual ABG parameters as predictors of mortality is rather counterproductive. The very assumption of multivariate regression analysis is that the variables in the study are independent [25]. We postulate that the use of multivariate regression analysis in order to evaluate the capacity of individual ABG parameters as prognostic markers is futile as the ABG parameters are not only interdependent on each other but some of these parameters are also derived from other ones. Thanachartwet et al. and Yacoub et al. have emphasized on the prognostic significance of serum lactate in dengue patient [20,24]. However, on multivariate regression analysis, we observed the failure of lactate levels as an independent prognostic marker in patients with severe dengue. Therefore, we propose that lactate can only be used as an additional alternative criterion in the prognostication of patients with severe dengue. Based on these, we propose basic ABG parameters and lactate as feasible prognostic markers in patients with severe dengue due to the conventional practice of their monitoring in every patient of critical care units.

We studied basic ABG parameters and lactate, at the time of admission to MICU in patients with severe dengue and found that lactate levels was the best (with maximum AUC on ROC analysis as compared to basic ABG parameters) predictor of mortality during intensive care stays in our study population. This is rather fortuitous for the following reasons: Firstly, lactate is an objective measure as opposed to the subjective warning signs proposed by WHO which have inherent variability in establishing not only their presence but also severity [9,14]. Secondly, the diagnosis of acid-base disorders is complex and thus requires expertise [5-7,13].

Under the assumption that the transfer of dengue patients to MICU due to critical illness (warning signs indicating predisposition to severe dengue according to WHO) is approximately the actual time of development of severe dengue, we believe that prognosticating at this point of time provides a reasonable approach [12,23]. We hypothesize that at this time, underlying pathophysiological processes that are related to poor prognosis would have reached a level of identifiable significance. Prognosticating prior to this point of time will jeopardize specificity, as the ultimate prognosis-determining processes may yet to occur. Prognosticating too late, however, may be nugatory as the crucial time for aggressive management in order to improved survival may have been lost [23]. However, it has to be noted that the underlying outcome determining pathophysiological mechanisms is yet to be clearly elucidated. Further investigation into the biochemical changes with respect to the timing of events in dengue and more importantly, at the time of development of severe dengue is needed.

Based on our observations, we propose that basic ABG parameters and lactate can be used as additional prognostic markers in patients with severe dengue. With the help of early prognostication, dengue patients at higher risk of mortality can be identified, and thus more informed decisions regarding intensive care of such patients can be made [22]. With the help of informed consensual decisions, discordant expectations regarding patient outcome (between patients’ attendants and health care professionals) can be prevented [1]. With timely interventions, not only patient management can be improved, but also judicious management of available resources is possible [2].

The strengths of our study are as follows: The main strength of our study is that it was well planned and executed in a state-of-the-art tertiary care hospital known for its adherence to institutional standard operating procedures and protocols. Further, well-defined inclusion and exclusion criteria, collection of arterial blood samples at a particular time in every study participant and more importantly the usage of the gold standard of WHO definition of severe dengue as inclusion criteria (rather than clinical terms like critically ill dengue patients as used in other studies aiming to evaluate prognostic markers in dengue) really add to its utility [10,11,16-24]. Another strength is that we studied the parameters that are advised as base line investigations in all critical patients and thus are readily available in every intensive care unit.

There are a few limitations of our study. The main limitation of our study is its retrospective design. However, we believe that the accuracy of the data is reasonable as we carried out arterial blood investigations at a specific time, i.e., at the time of their transfer to MICU and the management of all patients was also done following our standard treatment protocol. Our study included 163 subjects only. A study with a larger study population will provide results with more validity. Another limitation of this study was the measurement of all the biochemical parameters only once. Serial measurement of these parameters in mild and moderate states of the disease would not only provide a better understanding of the pathophysiological processes responsible for the development of severe dengue but would also help in prognosticating in regard to patients at the higher risk of development of severe illness [7,26,27]. Serum lactate, lactate clearance and base excess have emerged as important biomarkers with significant prognostic value in various clinical settings [26,27]. We couldn’t include results of base excess or deficit in the present study. Serial measurements of lactate levels for every 4-6 hours will give an idea about its clearance as well as its advantage as a prognostic marker over lactate levels at a particular time. Although we conclude that the lactate level at the time of shifting of patients with severe dengue to intensive care units is a good prognostic marker, we propose that serial lactate levels and lactate clearance could be a better predictor of mortality in these patients.

## Conclusions

Our study shows that basic ABG parameters and lactate at the time of admission to intensive care units are important predictors of mortality in patients with severe dengue. These parameters, especially lactate, may be used as feasible and utile prognostic markers in patients with severe dengue. The routine use of these simple, readily available, and cost‑effective parameters should be encouraged for prognosis, especially in diseases like dengue, as their clinical presentation may range from mild symptoms to severe life-threatening pathophysiological conditions.
